# In-season assessment of dietary intake and match demands of male collegiate rugby sevens players

**DOI:** 10.1080/15502783.2025.2550172

**Published:** 2025-12-17

**Authors:** Conor Horlock, Leah Allen, Margaret T. Jones, Andrew R. Jagim, Jennifer B. Fields

**Affiliations:** a University of Connecticut, Department of Nutritional Sciences, Storrs, CT, USA; b George Mason University, Sport, Recreation, and Tourism Management, Fairfax, VA, USA; c George Mason University, Frank Pettrone Center for Sports Performance, Fairfax, VA, USA; d Mayo Clinic Health System, Sports Medicine, Onalaska, WI, USA

**Keywords:** Nutritional needs, Athletes, Deficiency, Performance

## Abstract

**Background:**

The aims of this study were to 1) assess dietary intake of male collegiate rugby sevens players relative to sport dietary guidelines, 2) quantify match demands across a competitive season using GPS-derived workload metrics, and 3) explore changes in match workloads throughout tournament play.

**Methods:**

Division 1-AA male collegiate rugby sevens players (*n* = 13, age 20 ± 1 yrs; height 2 ± 0 m; weight 87 ± 14 kg; body fat 20 ± 4 %; FFM 71 ± 12 kg) participated. Dietary intake was assessed at the start of the season using a 4-day food log (MyNetDiary, Marlton, NJ, USA). Players were equipped with a 10-Hz GPS/GNSS device (Vector Core; Catapult Sports, Melbourne, Australia) to wear in all matches (*n* = 17) across four tournaments. GPS-derived workload metrics included: Total distance (m), sprint distance (> 20.1 km/hr), high-intensity distance (> 18.1–20.0 km/hr), meters per minute (m/min), acceleration efforts (#), deceleration efforts (#), sprint efforts (#), high speed efforts (#), player load (AU), player load per minute (AU/min), and estimated energy expenditure (kcal). A one-samples t-test was used to assess dietary intake for energy, carbohydrates, proteins, and fats relative to established recommended guidelines (40 kcal/kg bw, 5 g/kg bw, 1.6 g/kg bw, and 1 g/kg bw, respectively). A repeated measures analysis of variance examined changes in workload parameters across tournament matches (*p* < 0.05).

**Results:**

Players underconsumed calories (31.8 ± 10.6 kcal/kg, *p* = 0.028) and carbohydrates (2.8 ± 1.3 g/kg, *p* < 0.01) relative to recommended intakes. In contrast, protein intake was higher than guidelines (2.1 ± 0.7, *p* = 0.038), while fat intake did not differ (1.2 ± 0.4, *p* = 0.141). A descriptive summary of workload parameters and changes across matches is presented in Table 1.

**Table 1.** Summary of external workloads across tournament matches.

	Match 1	Match 2	Match 3	Match 4	Match 5	Match 6	Accumulated Tournament Loads	p-value
**TD (m)**	1105.96 ± 306.29	1094.47 ± 284.56	1074.08 ± 283.55	1235 ± 270.19	1313.91 ± 103.61	1106.94 ± 316.17	3861.69 ± 1649.96	< 0.001
**SD (m)**	32.03 ± 30.20	21.65 ± 26.78	18.79 ± 24.14	12.75 ± 22.26	20.93 ± 38.67	5.51 ± 8.79	71.89 ± 68.67	< 0.001
**HI distance (m)**	74.63 ± 57.02	46.85 ± 37.42	47.36 ± 43.82	61.18 ± 51.95	40.47 ± 40.32	47.04 ± 43.38	190.68 ± 162.54	< 0.001
**Meters/min**	68.44 ± 19.05	68.44 ± 19.05	67.13 ± 17.72	75.69 ± 15.92	82.12 ± 6.47	69.18 ± 19.76		< 0.001
**Accel (#)**	12 ± 6	11 ± 6	10 ± 6	12 ± 6	12 ± 6	11 ± 6	39 ± 23	< 0.001
**Decel (#)**	13 ± 6	12 ± 6	12 ± 6	14 ± 7	13 ± 5	12 ± 8	42 ± 20	< 0.001
**SE (#)**	1 ± 1	1 ± 1	1 ± 1	0 ± 1	1 ± 1	0 ± 1	3 ± 12	< 0.001
**HSE (#)**	4 ± 3	3 ± 2	3 ± 2	4 ± 3	3 ± 3	2 ± 2	12 ± 10	< 0.001
**PL (AU)**	117.83 ± 36.02	115.95 ± 31.89	110.55 ± 34.06	135.86 ± 33.50	141.10 ± 16.98	123.81 ± 34.92	411.71 ± 181.39	< 0.001
**PL/min (AU/min)**	7.28 ± 2.23	7.24 ± 1.99	6.9 ± 2.12	8.32 ± 2.00	8.81 ± 1.06	7.73 ± 2.18		< 0.001
**EE (kcal)**	260.50 ± 77.97	256.74 ± 71.63	248.81 ± 71.17	290.84 ± 68.50	304.45 ± 32.19	259.64 ± 79.49	903.91 ± 395.50	< 0.001

Values are presented as mean ± SD; TD = total distance; SD = sprint distance; HI distance = high intensity distance; meters/min = meterage per minute; accel = accelerations; decel = decelerations; PL = player load; PL/min = player load per minute; SE = sprint efforts; HSE = high speed efforts; EE = energy expenditure; AU = arbitrary units.

**Conclusion:**

Although players experienced high match workloads during tournaments, their energy and carbohydrate intakes were insufficient. This imbalance may compromise recovery and performance, underscoring the need for tailored nutritional strategies to meet the demands of competition.

1.

